# Dissection of lung parenchyma using electrocautery is a safe and acceptable method for anatomical sublobar resection

**DOI:** 10.1186/1749-8090-7-42

**Published:** 2012-05-03

**Authors:** Takashi Ohtsuka, Taichiro Goto, Masaki Anraku, Mitsutomo Kohno, Yotaro Izumi, Hirohisa Horinouchi, Hiroaki Nomori

**Affiliations:** 1 Department of Surgery, Section of General Thoracic Surgery, School of Medicine, Keio University, 35 Shinanomachi, Shinjuku-ku, Tokyo, 160-8582, Japan

**Keywords:** Pulmonary segmentectomy, Lung tissue sealing, Stapling devices

## Abstract

**Background:**

Anatomic sublobar resection is being assessed as a substitute to lobectomy for primary lung cancers. However, persistent air leak after anatomic sublobar resection is prevalent and increasing surgical morbidity and costs. The use of electrocautery is being popularized recently in anatomic sublobar resection. We have retrospectively evaluated the safety and efficacy of intersegmental plane dissection using electrocautery.

**Methods:**

Between April 2009 to September 2010, 47 patients were treated with segmentectomy for clinical T1N0M0 non-small cell lung cancers. The intersegmental plane was dissected using electrocautery alone or in combination with staplers. We evaluated the methods of dividing intersegmental plane (electrocautery alone or combination with electrocautery and staplers), intraoperative blood loss, duration of chest tube placement, duration of surgery, preoperative FEV_1.0_ %, incidence of prolonged air leak, length of postoperative hospital stay, postoperative pulmonary function at 6 months after surgery and the cost for sealing intersegmental plane.

**Results:**

Among the 47 patients, 22 patients underwent intersegmental plane dissection with electrocautery alone and 25 patients did in combination with electrocautery and staplers. The mean number of stapler cartridges used was only 1.3 in electrocautery and staplers group. Mean age, gender, number of patients whose FEV_1_% < 70 % were similar between two groups. There was no statistical difference between electrocautery alone and combination with electrocautery and staplers group in duration of surgery (282 vs. 290 minutes), intraoperative blood loss (203 vs.151 ml), duration of chest tube placement (3.2 vs. 3.1 days), postoperative hospital stay (11.0 vs.10.0 days), postoperative loss of FEV_1.0_ (13 vs.8 %), loss of FVC (11 vs. 6 %) or incidence of minor postoperative complications [9 % (2/22) vs. 16 % (4/25), p = 0.30)]. However, incidence of prolonged air leak was higher in electrocautery alone group than in combination with electrocautery and staplers group [14 % (3/22) vs. 4 % (1/25), p = 0.025)]. The cost of materials for sealing air leaks amounted to €964 per patient in the electrocautery alone group and €1594 per patient in combination with electrocautery and staplers group.

**Conclusions:**

The number of patients with prolonged air leak was higher in the electrocautery alone group. The use of staplers in addition to electrocautery may lead to reduced prolonged air leak. However, the use of electrocautery for intersegmental plane dissection appeared to be safe with acceptable postoperative complications and effective in reducing costs.

## Background

Segmentectomy is now going to be recognized as an acceptable procedure for early stage lung cancer [1]. However, persistent air leaks and intraoperative bleeding in the dissecting plane are common dilemmas for thoracic surgeons performing segmentectomy. Surgical staplers are widely used to dissect intersegmental plane for segmentectomy [2,3]. However, lung resection with staplers could result in lesser postoperative pulmonary function compared with resection without stapling devices, because of shrinkage of the preserved segment [4,5].

In this study, we retrospectively evaluated the feasibility and safety of intersegmental plane dissection using electrocautery.

## Patients and methods

This study protocol was approved by Keio University institutional review board (approval ID: 20-174). Written informed consent was obtained from each participant in accordance with the Declaration of Helsinki. Between April 2009 to September 2010, 47 patients were treated with segmentectomy with open thoracotomy for clinical T1N0M0 biopsy proven non-small cell lung cancers (NSCLC). All patients were operated by one of five surgeons specialized in thoracic surgery, and they were managed by the same team. Patients’ data was obtained included age, gender, smoking habits, site of resection and spirometry variables. The patients’ characteristics are summarized in Table 1. During segmentectomy, the intersegmental plane was identified with the procedure reported by Tsubota [6]. In summary, after the segmental bronchus was isolated, the whole lung was temporarily inflated. The segmental bronchus was first ligated to retain the air inside the segment and then divided at the point of proximal to the ligation. Single lung ventilation was initiated, producing the inflated – deflated line between the resecting segments and preserving ones. The intersegmental plane was then dissected along the inflated – deflated line using mainly electrocautery alone. Surgeons were allowed to use the staplers in dissecting thick lung parenchyma, but were enforced to use as little as possible. Electrocautery output was set for 60 watts. During the procedure, the surgeon retracted the segment to be removed with one hand, and used electrocautery with the other hand. The decision of using staplers in addition to electrocautery was made by the surgeon. After the dissection of intersegmental plane, the plane was covered with a fibrin sealant, composed of fibrinogen and thrombin and an absorbable polyglycolic acid felt (Neoveil; Japan Medical Planning Co, Kyoto, Japan). Afterward, all patients received single chest tube drainage (28Fr) and were connected to Chest Drainage Vac (Sumitomo Bakelite, Tokyo, Japan). Chest tubes were placed to water seal after surgery. To remove the tube, the volume of drained fluids was required to be less than 200 mL during the preceding 24-hour period and all the leaks resolved. We evaluated the methods of dividing intersegmental plane (electrocautery alone or combination with electrocautery and staplers), intraoperative blood loss, interval between surgery and chest tube removal, duration of surgery, preoperative FEV_1.0_ %, number of patients with prolonged air leak more than 7 days, length of postoperative hospital stay, pneumothorax after the chest tube removal and the cost of materials for sealing air leaks.

**Table 1:** Characteristics of patients

		Electrocautery	Electrocautery and staplers
Number		22	25
Gender	Male	9	13
	Female	13	12
Age		62 ± 10	66 ± 11
Smoking history	Yes	14	14
	No	8	11

### Statistical analysis

The unpaired Student’s *t* test was used to test relationships between discrete variables and continuous variables. The χ -square test was used to compare discrete variables.

## Results

Among the 47 patients, 22 patients underwent intersegmental plane dissection with electrocautery alone and 25 patients underwent intersegmental plane dissection in combination with electrocautery and staplers. The two groups were absolutely similar when sex, age, and smoke exposure were compared. There were no intraoperative complications and no perioperative deaths. Table 2 shows the locations of burdened segments. The comparisons of the two groups are shown in Table 3. There was no difference in two groups in number of patients whose FEV_1_% < 70 %. Duration of surgery was 282 ± 71 min in electrocautery alone group, and 290 ± 64 min in combination with electrocautery and staplers group ( p = 0.695) . Number of staplers used was1.3 ± 0.7 (range1-4) in combination with electrocautery and staplers group. Duration of chest tube placement was 3.2 days in electrocautery alone group and 3.1 days in combination with electrocautery and staplers group (p = 0.957). There was no difference in two groups in occurrence of pneumothorax after chest tube removal, and hospital stay. However, the number of patients with prolonged air leak more than 7 days was higher in the electrocautery alone group than that in combination with electrocautery and staplers group (3 and 1, respectively, p = 0.025). Postoperative complications developed in 2 patients (9 %) in electrocautery alone group and 4 patients (16 %) in combination with electrocautery and staplers group (p = 0.31) (Table 4.). There was no perioperative death. The postoperative loss of FVC and FEV_1.0_ was 11 % and 13 % in electrocautery alone group and 6 % and 8 % in combination with electrocautery and staplers group (p = 0.48 and 0.30, respectively) (Table 5). The mean cost of materials for sealing air leaks including the cost of staplers amounted to €964 per patient in the electrocautery alone group and €1594 (range 1495 to 2421) per patient in the electrocautery and staplers group (p < 0.01).

**Table 2:** Location of burdened lung

		Electrocautery	Electrocautery and staplers
Lobe			
	Right upper	3	11
	Right lower	5	5
	Left upper	8	7
	Left lower	6	2
Resected segment(s)			
Right	S1a + 2	2	
	S2 + 3a	1	
	S2b + 3a		1
	S2		3
	S1b + S3		1
	S1		4
	S1 + 2		1
	S3		1
	S8		3
	S6	4	2
	S8 + 9	1	
Left	S1 + 2	5	1
	S1 + 2 + 3	2	4
	S4 + 5	1	2
	S6	2	1
	S8	1	
	S8 + 9	1	
	S9 + 10	2	1

a, posterior subsegment; b, anterior subsegment; S1, apical; S2, posterior; S3, anterior; S4, superior; S5, inferior; S6, superior; S8, anterior basal; S9, lateral basal; S10, posteiror basal.

**Table 3:** Comparison of patients

	Electrocautery	Electrocautery and stapler	*p* value
Number of patients whose FEV1% < 70 %	8	10	0.495
Duration of surgery (min)	282 ± 71	290 ± 64	0.695
Duration of chest tube drainage (days)	3.2 ± 3.0	3.1 ± 4.0	0.957
Duration of hospital stay after surgery (days)	11.0 ± 4.6	10.0 ± 4.7	0.450
Number of patients with prolonged air leak more than 7 days	3	1	0.025
Number of patients with pneumothorax after chest tube removal	1	3	0.240
Intraoperative bleeding (ml)	203 ± 214	151 ± 116	0.305

**Table 4:** Postoperative complications

	Electrocautery (n = 22)	Electrocautery and staplers ( n = 25)
Atelectasis	0	2
Pneumonia	1	1
Arrhythmia	1	1
Total	2	4

**Table 5:** Postoperative pulmonary function change

	Electrocautery	Electrocautery and stapler	p value
Postoperative FVC / Preoperative FVC	0.89 ± 0.20	0.94 ± 0.21	0.48
Postoperative FEV_1.0_ / Preoperative FEV_1.0_	0.87 ± 0.14	0.92 ± 0.18	0.30

FVC, forced vital capacity; FEV_1.0_, forced expiratory volume in 1 second.

## Discussion

In recent years and there is growing evidence to suggest that segmentectomy can yield results equivalent to lobectomy in patients with early stage peripheral lung cancers [1,7]. Moreover, several studies showed segmentectomy offers significantly better functional preservation compared with lobectomy [8,9]. Indications of segmentectomy as a treatment option for early stage lung cancer are spreading. Staplers are widely used for intersegmental dissection because of reliable hemostasis and easiness in use. However, intersegmental plane dissection with staplers could cause shrinkage of the preserved segment. Asakura et al. showed staplers interfere the expansion of preserved lung in comparison to scissors in swine lung segmentectomy model [5]. Additionally, staplers divide the intersegmental plane without recognizing the intersegmental veins, which are important for the drainage of the preserved segment. Sacrificing the pulmonary vein could cause an impairment of gas exchange leading to decrease in pulmonary function. Although intersegmental dissection with electrocautery has been thought to increase the incidence of postoperative air leaks and intraoperative bleeding, our results demonstrated 14 % of prolonged air leaks and 203 ml of intraoperative bleeding in the electrocautery alone group, which were similar to the incidence reported by others performed segmentectomy using staplers [2,3]. In addition, the combination use of staplers with electrocautery showed acceptable morbidity and minimum postoperative loss of respiratory function.

Drogehetti et al. showed that the use of electrocautery and collagen patches reduced the incidence of air leaks, duration of air leaks, and procedure costs compared with the use of staplers in dissecting interlober fissures [10]. However, there have been few reports evaluating intersegmental plane dissection with electrocautery. Our report here is the first article evaluating the usefulness of intersegmental plane dissection with electrocautery.

Our study has several limitations including its retrospective nature and small number of patients. In addition, the use of fibrin sealant and absorbable polyglycolic acid felt can make the comparison of the dissecting method unclear. However, we believe the results of this retrospective analysis of intersegmental dissection using electrocautery confirm that this technique can be performed with acceptable morbidity.

## Competing Interest

The authors declare that they have no competing interests.

## Authors' contributions

TO conceived of the study, participated in its design and coordination, drafted the manuscript and performed the statistical analysis. All authors have read and approved the final manuscript.
